# The Hospital. Nursing Mirror

**Published:** 1898-08-27

**Authors:** 


"The Hospital" Nursing Mirror.

Being the Nursing Section of "The Hospital."

[Contributions for this Section of "The Hospital" should be addressed to the Editor, The Hospital, 28 & 29, Southampton Street, Strand, London, W.C., and should have the word "Nursing" plainly written in left-hand top corner of the envelope.]

### News from the Nursing World.

### THE PRINCESS HENRY OF BATTENBERG AT GOSPORT.

On Saturday last the Princess Henry of Battenberg, accompanied by Miss Minnie Cochrane and Lord William Cecil, attended a public meeting of the supporters of the Victoria Society for Nursing the Sick Poor of Gosport, of which she is the president. The Princess crossed from Cowes in the Royal yacht "Alberta," and on reaching Thorngate Hall, Gosport, was received by a guard of honour of the local Volunteers, and conducted to the platform by Deputy-Inspector Woods, C.B., of the Royal yacht "Victoria and Albert," where she was presented with a bouquet by Miss Dorothea Brodrick. After a resolution pledging the meeting to support the society, the Princess distributed the certificates and medals to the members of the St. John Ambulance Association. Canon Brodrick proposed and Dr. Sykes seconded a vote of thanks to the Princess for her presence, and then everyone withdrew to an adjoining meadow to witness a demonstration of skill by the ambulance members, a mimic fight by the volunteers, and a display by the fire brigade, at the conclusion of which the Princess expressed herself as much pleased with the arrangements, and accepted, as a memento of her visit, a collection of views in the neighbourhood from Mrs. Cooke, wife of the chairman of the District Council.

### CHANGES AT THE LONDON TEMPERANCE HOSPITAL.

Universal regret is expressed on all sides at the resignation of Miss Orme, who for 25 years has been the lady superintendent (or matron, as she prefers to be called) of the London Temperance Ho pital. Miss Orme has taken charge of the nursing and all the multitudinous duties that cluster round the office of matron since the time the hospital was situated in Gower Street and had only sixteen beds. She has seen it grow on its present excellent site, bit by bit, until it has become a handsome and well-constructed building of 120 beds, the last acquisition being the nurses' home recently opened. On October 3rd, the twenty-fifth anniversary of the opening, Miss Orme proposes to bid farewell to her charge though not altogether to her hospital, for that lies very near her heart, and she will ever take the keenest interest in its welfare. Her reasons for doing so are well advised; she prefers to lay aside an onerous though beloved work before it proves beyond her powers. After a prolonged holiday she intends taking up some congenial but lighter task, not yet chosen, which will give her leisure and opportunities for enjoying social intercourse, impossible in a busy official life. A short while ago the hospital lost the services of Sister Wilson, of the "Sturge" and "Children's" wards, who had been in the hospital for 12 years. Miss Wilson has just married Dr. W. J Collins, the visiting surgeon. It is certain then that the institution will retain her friendship, which she has hitherto exercised in a very generous way. Amongst other things she was the donor of the furniture in the new aseptic ward.

### THE IRONY OF CERTIFICATES.

Two ladies, Sisters of the Dominican Order, were recently awarded the Royal Red Cross for distinguished nursing services, extending over a period of seven years. These ladies, Mother Jacoba and Mother Patrick, have arrived in Dublin to train for their certificates, as they desire to continue their good work in Rhodesia, and it has very rightly been decreed that all nurses in the Rhodesian hospitals must in future be fully qualified and certificated. It seems to us that a seven years' service amidst all the obstacles and trials attending the founding of a new colony, service recognised as efficient by our Queen, might well be accepted as a qualification. The Sisters may not be acquainted with all the latest scientific nursing appliances, but they have proved their worth for all that. We trust that the Sisters will not be hardly treated in the examination-room, for it is a well-known fact that the "hare" in theory is not infrequently the "tortoise" as regards practice, and that hard practical work is not the best preparation for mental effort.

### THE CEYLON NURSES' ASSOCIATION.

On July 21st the annual meeting of the Ceylon Nurses' Association was held at the Adam's Peak Hotel, Hatton. An inducement was offered to assistant superintendents to join the association by reducing their subscriptions to a minimum of five rupees. The matron's salary will in future be increased by granting her a capitation fee of five rupees a week on all cases coming into the wards. The question of massage was discussed, as the matron is not prepared to continue it, and it is doubtful whether or not any European nurse can stand the strain in the climate. The committee were determined not to give up this treatment, and debated the advisability of training a native woman for the work, or of employing in this direction another nurse to be supplied by the Colonial Nursing Association. The matter resolved itself into the hon. secretary being empowered to make what arrangements she thought best, and to bring them before the executive committee for confirmation at the next meeting. Nurses are allowed grants towards their homeward passages at the end of three, four, five, and six years' service. The grant at the end of five years' work, which had not been previously stated, was fixed at £20. As several of our correspondents have asked lately about the possibility of obtaining work in India, the address of the Colonial Nursing Association may be useful. It is the Imperial Institute, W.

### ANOTHER TYPHOID EPIDEMIC.

Belfast is in the throes of a typhoid epidemic which is assuming alarming proportions. The number of sick is rapidly increasing, and in the workhouse hospital alone there are 300 cases. The Board of Guardians has decided to advertise for two additional doctors and twelve nurses. The nursing arrangements of the workhouse have very recently been revised, and are by no means adequate for the severe strain put upon them, which has been so great that several of the nurses have broken down under it.

### ACCIDENT TO A PLAGUE NURSE.

The need of institutes in India where M. Pasteur's treatment for persons injured by animals suffering from rabies can be carried out is unfortunately brought closely home by the fact that Miss Harris, one of the lady nurses on plague duty in India, has been bitten by her own pet dog. The animal became ill after having been bitten by a stray cat, and, in endeavouring to secure it, the nurse was wounded severely in the hand. The next day the dog died from rabies. Miss Harris was advised to proceed at once to Paris, and we trust that, in spite of the ten days' journey, she may successfully escape the terrible consequences possible after such an accident. It appears that rabies and hydrophobia are prevalent in India, and it is such incidents as this that reconcile all but the constitutional grumblers in England to the steady enforcement of the muzzling order, which is practically stamping out a cruel and perfectly preventable disease.

### NOTABLE AMERICAN WOMEN.

Two American ladies attract considerable notice at the moment; they are Miss Margherita Hamm and Miss Cecil Charles. Miss Hamm has been appointed supervisor and inspector of supplies, and head of the nurses' staff in the Red Cross department of the National Guard, U.S.A. She is 27 years of age, a French Canadian by birth, and is regarded as "immune," having survived the fevers of the tropics. She is a great traveller, and has rendered good service as nurse in the war between China and Japan, and during the plague season at Hong Kong. Her portrait in the *Queen* of the 20th inst. shows a resolute, alert face. Miss Charles is also considered "immune." She is a Spanish-American, a Cuban patriot, and was one of the first volunteers to the Red Cross Society for work in Cuba. As an author she has employed her pen in pleading for a free Cuba during the last seven years.

### THE NURSES' HOSTEL.

Miss Wood, the founder and managing director of the Nurses' Hostel, is away holiday making. It is just over a year ago that the new building was opened, and the shareholders will soon be in possession of their first report. Miss Nancy Paul, the secretary, informs us that "the hostel appears to have fully met the want it was intended to supply, and has been of service to nurses from all parts of England and the colonies."

### DEATH OF A VETERAN NURSE.

Another veteran nurse, who won her laurels in the Crimea, has just died at Edinburgh. She was the young widow of one of the soldiers killed at Alma when Miss Florence Nightingale enlisted her services in the care of the sick and wounded at Scutari, and when she left that hospital she carried with her a testimonial from Miss Nightingale herself, saying how much the writer had appreciated her womanly sympathy and watchful care of the sick. Mrs. M'Nichol, for such was her name, married a second time, a comrade of her first husband, Mr. Samuel Evans, V.C., who survives her. The Patriotic Fund allowed her a pension of eightpence a day for a time, when it was reduced to fourpence. Fortunately she was permitted to keep this until her death.

### GARSTANG AND ITS NEIGHBOURHOOD.

Garstang and the adjoining districts have a flourishing little nursing association. Nurse Dell, helped by assistants, has been busy lately, and the night work has been heavy. The nurse has a salary of £80 a year and residence. A pony carriage is kept for her use, but the committee would probably find that a bicycle was cheaper, as the keep of the pony for one year would not only buy the cycle but keep it in repair for a long while. The honorary officers are to be congratulated on the increase of subscriptions and donations, and on the fact that the value of the pony, which died during the year, was replaced twice over. All this shows that the nurse is popular, and possesses the sympathy of the public.

### SHORT ITEMS.

Probationers on staff duty at the London Hospital wear a silver "S" on the sleeve; the senior probabationers at Guy's Hospital are distinguished by a narrow white band on the sleeve four inches from the wrist; and the nurses possessing the three-year certificate at St. Bartholomew's wear a blue belt.—The *Indian Daily News* is responsible for the statement "that the nurse engaged for special plague duty from the middle of May was relieved on July 15th by a nurse just arrived from Bombay, and has been dismissed without notice. Her work—inspecting the female passengers on vessels before entering port—was satisfactory, and promptly done, yet nothing of her salary for the whole period of her employment has yet been paid up to Friday" (July 22nd).—The Visiting Committee of the Brighton Workhouse Infirmary recommend that the Local Government Board be asked to reconsider and sanction the appointment of Mrs. Burden—the present matron—as superintendent nurse. Mrs. Burden has discharged the duties of the latter office to the satisfaction of the committee since her appointment in 1892.—The Tiverton Nursing Association's gymkhana realised £68. —The tenth annual report of the Liskeard Town Nurse is published. The nurse has attended 97 cases, some of them chronic, and the outlay has been £48. It is therefore, very satisfactory to see that her committee wish to increase her salary; the difficulty is, of course, to find the money. There is a deficit in the year's working expenses, which, however, is more than covered by the balance in hand at the commencement of the year.—The National Health Society is making arrangements for the winter classes. There will be a series of lectures for ladies preparing as sanitary inspectors and lecturers. Apply for particulars to the Secretary, 53, Berners Street, W.

### Antiseptics for Nurses.

By a Medical Woman.

### XV.—NAPHTHOL AND ITS COMPOUNDS AS ANTISEPTICS.

Naphthalene in its pure state is a white crystalline solid having a greasy feel to the touch and a distinctly tarry odour. It is very slowly volatile, quite insoluble in water, and has only feebly disinfectant properties. It is largely used as a foundation for certain cheap patent blocks and cakes which are sold as "disinfectants," and are strongly recommended by the inventors for hanging up in closets and over sinks with a view to purifying the air. Since these patent blocks contain little else, as a rule, but earthy matters and a small amount of naphthalene, which, as we have already seen, is only slightly volatile and disinfectant, the futility of depending on such for disinfection or even for deodorisation is at once evident. Some kinds of similar blocks are said to contain camphor and eucalyptus in addition to the naphthalene, and the presence of these additional and very volatile antiseptics certainly increases their value as deodorants, but under no circumstances can they be regarded as more than this, nor can they lay any real claim to being efficient disinfectants. They have, however, proved to be of some use as insecticides.

Naftalan is one of the many preparations obtained from the petroleum springs of the same name in the Caucasus. It is a thick very dark green fatty substance having a slightly empyrumatic odour. Like naphthalene, it is quite insoluble in water and in glycerine, but is soluble in ether, chloroform, and, less so, in alcohol. It mixes very readily with vaseline, lanoline, or any other fatty substance. It is said to be a harmless but nevertheless good antiseptic, and has, in addition, a slightly sedative action. It has been used more extensively on the Continent than in this country, and Dr. Rosenbaum reports of it as possessing marked antiseptic properties, and is convinced of its efficiency in "inflammatory processes and in certain skin affections. It has a vigorous deodorant action in gangrenous processes; the foul smell was rapidly subdued, and healing took place in a very short time. This agent is of great service in frostbite of the extremities," says the same authority," as it produces a hyperæmia of the part, and wards off threatening gangrene. Here, also, as in burns, naftalan exercises an anodyne action."

Naphthalene sulphonic acid is produced by adding strong or concentrated sulphuric acid to naphthalene, and applying heat to the mixture, the result of this is a crystalline deliquescent substance which is readily soluble in water, is strongly acid and almost inodorous. The next step in the preparation is to dissolve the above in water previously saturated with chalk, and it is allowed to crystallize. If carbonate of soda is subsequently added to the mixture before crystallization has occurred, it is thus converted into a sodium salt, and the substance known as naphthalene sulphonic acid is obtained. It has occasionally been used as a disinfectant, but it is troublesome to prepare, and its disinfectant powers being very feeble, it has now practically gone out of use. Each of these two forms of naphthalene sulphonic acid, viz., that prepared with chalk and with carbonate of soda respectively, has been fused at a moderate temperature with caustic soda, and then yields the corresponding naphthol, which is subsequently purified by pressure, distillation, and crystallization from hot water or petroleum ether. One of these, which is called beta-naphthol, is well-known commercially. It crystallizes in lustrous plates which are colourless when pure and should not darken when exposed to light, which smell like phenol and have a hot, tarry taste; they should be neutral in reaction and leave no ash when burnt on platinum wire. Beta-naphthol is almost insoluble in cold water, but dissolves rather more readily in boiling water. It is soluble in rectified spirit, ether, chloroform, olive oil, and glycerine. It is a powerful antiseptic, and can be used either as an ointment or in solution, and is strongly recommended by Professor Bouchard for external use. If naphthol and salt be rubbed together they form a fluid, but beta-naphthol alone does not liquefy. It does, however, assume a permanently fluid form when melted with camphor, giving a substance known as camphorated beta-naphthol.

Betol, or naphthol salicylate, is a white crystalline inodorous and tasteless powder, neutral in reaction, insoluble in water and in glycerine, soluble with difficulty in alcohol and turpentine; more readily in hot alcohol, in ether, and in warm linseed oil. If hot acids and alkalies be added, it is reconverted into salicylic acid and beta-naphthol. It combines the antiseptic properties of its two constituents, but is now very rarely used.

Hydro-naphthol is another of the naphthol series, and is prepared by the reduction of beta-naphthol by sodium. It occurs as a whitish powder of very fine, flaky, shining crystals, having an odour somewhat like creosote. The powder is only very slightly soluble in cold water, somewhat more so in hot water to the extent of one part in 2,000; but after cooling there is also a considerable layer of insoluble hydro-naphthol in the solution. It is, however, readily soluble in alcohol, and Dr. Bryce strongly recommends the following as the best preparation, viz.: Hydro-naphthol, one part; rectified spirits of wine, 10 parts; glycerine, nine parts. This makes a brownish fluid of the strength of one part of 100; and in this form its antiseptic properties are well marked; but although hydro-naphthol has been proved by repeated and careful experiments to be a powerful antiseptic, its insolubilityin water will prevent it being widely used. It is said to be non-poisonous, non-corrosive, and non-irritant. A 5 per cent. hydro-naphthol gauze is prepared, and is recommended as "very lasting and as compatible with albuminous fluids."

Hydroxy-naphthol acid is also know by the name of oxy-naphthol acid. It is prepared by the action of carbonic acid gas on a saturated solution of sodium naphthol. Like all the other napthol preparations, this one is also sparingly soluble in water, more easily so in alcohol, in ether, and in solutions of the alkalies. Collodion impregnated with ½ per cent. of hydroxy-naphthol acid is recommended as an excellent substitute for iodoform collodion, as it is non-irritating and more stable. The various salts of hydroxy-naphthol acid are not antiseptic, and it is very doubtful whether they are innocuous.

Alumnol is formed by heating any of the naphthols with concentrated sulphuric acid, and then adding a saturated solution of aluminium hydroxide. This gives an aluminium salt, which is known by the name of alumnol, and which contains 5 per cent of aluminium. It is a white stable powder, which is very soluble in water, and is also soluble in alcohol, but with a blue fluorescence. It dissolves in glycerine, but not in ether. Its reaction is slightly acid, with albumen and gelatine alumnol forms a white precipitate, which is, however, readily redissolved in excess. Alumnol has no injurious effects if it is employed judiciously; it causes neither pain or irritation. It is a soluble, non-toxic, astringent, strongly penetrating, and antiseptic compound. Dr. Spengler, of Heidelberg, has used alumnol extensively and considers it "quite equal to chloride of zinc, whilst it has the great advantage of not being in the least unpleasant to use."

Menthoxal, also known as camphoxal and naphthoxal, consists of a hydroxyl solution which contains respectively menthol, camphor, or naphthol, to which alcohol is added. These substances are little known, and are of little, if any, value as antiseptics.

Hydro-naphthol pastilles are in the form of cones, and ignite very easily. As they burn hydro-naphthol is liberated and diffused through the air. The odour is agreeable and is very useful as a deodorant in a sick room.

### Training in the Provinces.

(*Continued from page* 181.)

### GUEST HOSPITAL, DUDLEY (100 Beds).

### Terms of Training.

Candidates for training should be between 20 and 35 years of age. A four weeks' probation is required before a definite engagement is entered into, and if a candidate leaves the hospital before the expiration of this period she is charged 10s. 6d. a week in payment for board, lodging, and washing. If a candidate is approved she may be received for two years' training, paying a premium of twenty guineas, a sum which, except under special circumstances, will be forfeited if the engagement is broken. Courses of lectures are given by members of the medical staff during the period of training, and general instruction in nursing by the sisters of the wards, a certificate of efficiency being granted, if deserved, at the end of the two years. At the end of the two years nurses may either join the private nursing staff, if there is a vacancy and they are suitable, when they receive a two years' certificate, with a salary of £20 and part uniform; or they may remain in the service of the hospital for one year's further training, in which case they are paid a salary of £10 and are provided with part uniform, receiving a three years' certificate. Sisters of wards may be selected from those nurses who have gained a three years' certificate at the Guest Hospital, or they may be appointed from outside. In any case the three years' certificate is essential as a qualification.

The salaries of staff nurses, or third year probationers, range from £10 to £20; sisters' salaries vary from £25 to £35, according to the size and nature of their wards.

There is a night sister, and night duty is taken by probationers for two consecutive months in alternation with four months' day duty.

For the first two years probationers are required to provide themselves with both indoor and outdoor uniform.

### Hours On and Off Duty.

Nurses and probationers are on duty in the wards from 7 a.m. to 8.30 p.m., with the exception of time at appointed hours for meals and two hours daily for recreation at such time as may be convenient. It is expected that at least half an hour of this time shall be spent in the open air. On Sunday every nurse and probationer has a half-day off duty, either from 10 to 2 or from 2 to 9.30, and four weeks' leave is given during the two years' training. The sisters go on duty an hour later than the probationers in the morning. They are off duty every other day from 6 to 9.30 p.m., with a half-day once a week and every Sunday from 10 to 2 or 2 to 9.30 p.m.

Night nurses are on duty from 8.30 p.m. to 8.30 a.m., taking their recreation time between their 9.15 a.m. dinner and 12.30, when they are expected to be in bed. Their rules require them to go out for half an hour every day. All the nursing staff are required to be present at Divine service at least once every Sunday.

### Meals.

Nurses and probationers' time-tables are as follows: Breakfast, 6.40 a.m.; lunch, 9.30 a.m.; dinner, 12.30 p.m.; tea, 4 p.m.; supper, 8.40 p.m. Sisters breakfast at 7.40, lunch between 9.30 and 10 a.m., and dine at 1.30 to 2.15 p.m. Their tea and supper are served in their private sitting rooms. The nurses' meals, with the exception of lunch, are taken in the dining-room.

Night nurses breakfast at 8 p.m. and dine at 9.15 a.m.; their night meals are served in the ward sitting-rooms, where the day nurses also take their lunch. Provisions for these meals, coffee, tea, sugar, butter, are given out, the three former weekly, the latter daily.

### DEVON AND EXETER HOSPITAL (214 Beds).

### Terms of Training.

Probationers are received at the Devon and Exeter Hospital for four years' training, a limited number of paying probationers being received for one year for a premium of 15 guineas. Candidates should be between 23 and 35 years of age. Ordinary probationers receive no salary for the first six months; then they are paid £10 per annum until the examinations have been satisfactorily passed; after this they receive £20 a year until three years are completed. Nurses on the hospital staff then receive a salary of £25 per annum; nurses on the private staff begin at £25, increasing £2 10s. per annum to £30. Sisters' salaries begin at £30, increasing £2 10s. per annum to £35. All are provided with board, lodging, washing, and medical attendance, and material for uniform dresses and caps. No certificate is granted until the end of the four years' training, and then only to such probationers as present a satisfactory report of their work in the wards and have passed the examinations. Probationers are required during their training to attend the lectures and classes.

All probationers are alike eligible for promotion to staff appointments on their merits; the sisters are appointed after three years' training according to suitability. After the first year probationers take day and night staff work, the night nurses being appointed for six to ten months at a time.

### Hours On and Off Duty.

Probationers go on duty in the wards at 7 a.m., and leave them at 8.30 in the evening. From this an hour and a half must be deducted for meals, and two hours daily for recreation. Probationers are off duty either from 10 to 12 in the morning, or from 2.30 to 4.30 in the afternoon. They have also every third Sunday from 2.30 to 9 p.m., and one day a month from 10 a.m. to 9 p.m. Staff nurses are on duty between 7 a.m. and 9.30 p.m., deducting two hours for meals and three hours to three hours and a quarter for recreation. They go off duty from 2.45 to 6 p.m., or from 6 to 9.30 p.m.; they have also from 2.30 to 9.30 p.m. every alternate Sunday, and one day a month from 10 a.m. to 9.30 p.m., as the state of the wards permits. Night nurses are on duty from 9.30 p.m. to 8.30 a.m., with half an hour off during the night for a meal. Sisters go on duty at 8 a.m., and are off duty daily from 2.45 to 6 p.m., or from 6 to 9.30 p.m.; on every alternate Sunday from 2.30 to 9.30 p.m., and one day a month from 10 a.m. to 9.30 p.m. as the work permits. Three weeks annual holiday is given to the nurses, and 28 days to the sisters during the year. Sisters and nurses are on duty until ten a.m. on their days off.

### Meals.

The day nurses' breakfast is at 6 30 a.m., at 9.30 half an hour is allowed for dressing and lunch, dinner is at 12.30 or 1.30, tea 4 to 5, supper 8 and 8.30 p.m. Sisters breakfast at 8.15 a.m. and dine at 1.30 p.m. Lunch and afternoon tea are served in their own rooms. The nurses take their lunch in the ward kitchens. Tea and all other meals are laid in the nurses' dining room. The night nurses breakfast at 9 p.m. and dine at 9.15 a.m. Butter, sugar, and tea are given out to the sisters once a week; butter is also given out to the nurses.

### Wants and Workers.

Dr. Dobdge, Glastonbury, Somerset, would be glad of any information as to the rules and management of a nursing home for a district nurse which has also a small emergency ward.

Nurse Hunt, Ollerton, near Newark, would be glad to hear of a second-hand invalid Reading tricycle for sale.

### Nursing in Paris Hospitals.

### C.—THE NURSING SISTERS.

### II.—Recruitment, Training, Regulations.

The nursing Sisters, as everyone knows, are recruited from the most exalted religious devotees of the Catholic community, and are the counterparts, as I have before had occasion to remark, of the women who in Protestant countries devote themselves to nursing work from a desire to lead lives of devotion rather than from mere search after a living. The feeling of religious exaltation among Catholic women impels them in two widely different directions: If the feeling be merely a dreamy spiritualism, it leads to the seclusion of the nunnery; if the religious impulse is coupled with deep human sympathy, it points to the lowly but noble drudgery of the Sister of Charity. The Sisters come from all ranks in life, from princesses of bluest lineage to peasants of humblest origin. The French Sisters who have during the last fifty years received the august tribute of the Legion of Honour included as the two most noted examples just this contrast: the Sister Rosalie, a peasant born, and the Sister Natalie Narischkin, a princess by birth and breeding.

The Sisters' system of training is perhaps one of the most admirably arranged for effective selection of the fittest for each post. Being under vows of strict obedience, and bound to do whatever is allotted them, with no personal positions to make, the novices are placed under the care of an experienced nurse and have their capacities tried. When the novice is found to be impossible material out of which to make a really trustworthy nurse for the bedside she is relegated to the linen-room, the kitchen, or some other function, and only the really capable are retained in the regular course of nursing, until they become competent to direct others.

The routine of the daily service of the Nursing Sisters is set forth in their rules which read in substance as follows: "Arise at 4 a.m.; in wards from 5 a.m. to 6 a.m. on duty, overseeing distribution of first meal to patients, house cleaning operations; at 6.30 a.m., mass, rest; breakfast at 7. Sisters return to the wards at 7.30, staying until 12.30 p.m., overseeing distribution of medicaments and the 10 o'clock repast to patients, and taking charge of the ward during the meal and during the rest and absence of male and female nurses; at 12.30 p.m., lunch, rest; 2 p.m. to 6 p.m., service in wards, including distribution of 4 p.m. meal and medicaments prescribed during day; 6 p.m. to 7.15 p.m., prayers, dinner, and rest; 7.15 p.m. to 8.30 p.m., visits to wards, distribution of night medicaments. For night service a sister watches and visits the wards first between 10 p.m and 12.30 a.m., and second visit between 1 a.m. and 3 a.m. The Sisters of a ward are at all hours of the night at the first call, always at the disposition of the visiting physician, the house surgeon, the director, the steward, &c."

Of course, these rules are somewhat varied according to the habits of the different orders of Sisters. It is well to remark, in connection with the religious observances of the Sisters, that there is a radical difference between the Sisters of Charity and the other Sisters. The Daughters of Saint Vincent de Paul are not properly nuns at all. They have no permanent vows. On the old style New Year's Day, every 25th of March, their individual obligations are renewed. This is, however, something like the constitutional figment that the British army has no permanent existence, but has to be annually renewed by the voting of the Army Bill. The Sisters of Charity consider themselves more permanent institutions than does even Tommy Atkins himself. They, nevertheless, do not look upon themselves as nuns, only women living in common for charitable work, foregoing domestic relations it is true, but not under religious vows. Although, of course, very unusual, and probably with much reprobation if so done, yet a Daughter of Charity can abandon her calling without incurring any penalties of Church discipline.

This peculiar point about the Daughters of Saint Vincent is worth laying stress upon in connection with the frequent allegations of the laicisers that the nursing Sisters devote too much time to religious duties, and also misuse opportunities at the bedside of patients in proselytising work. As the Daughters of Charity are thus shown to be, after all, only laics themselves, and the other chief Order in the Paris hospitals is the Augustinians of the Hôtel Dieu, which was only organised for this nursing work, the charge seems in a great measure uncalled for, if it has any reason at all.

The hours above indicate the very limited amount of time taken by the Sisters in the daily orisons, while as an offset for this, and also for any extra time occupied at the bedside in religious discourses, we must remember that the Sisters take no holidays nor leaves of absence like the laics, but keep to their posts year after year without intermission, or, at least, so little as to not be considered; in fact, one of the hospital physicians, in a letter I shall quote latter on, in treating of the question of professional esteem of the Sisters, instances the case of a Sister who applied for a short leave to visit her relations in the country, on the plea that she had not been absent for 25 years, and had seen none of her people during that period. This is a most striking example of devotion to duty for that specimen of well known ne'er-do-well who is always applying for "days off" to christen, to marry, or to bury imaginary kinfolk.

It is a curious fact that a complaint of Cardinal La Rochefoucauld, over two centuries ago, that the nurses at La Pitié belonged to no congregation, has been quoted in support of the antiquity of laicisation. Perhaps these irregular Sisters were merely voluntary disciples of the new teaching of St. Vincent, who told his Daughters of Charity to be "daughters of the parish" where they resided, and do the most needful good work at hand. I cannot see that, as they say in parliamentary practice, "the point is well taken." Altogether, both in the past and to-day, most evidence points to the fact that the Sisters attend to their business of nursing before anything else.

As a matter of regulations for the Sisters I will simply mention that they are allowed a salary of 200 francs a year, besides their keep, which, of course, with them goes into a common fund. The question of pay is, of course, more important in dealing with comparative cost, which I shall treat of in the next article but one from this. Edmund R. Spearman.

### Our American Letter.

An important article appears in the August number of the *Trained Nurse*, entitled "The Training School of the Future," from the pen of Miss Helena McMillan.

From it we learn that the nursing profession is sadly overcrowded in America, especially in the eastern States, and that the causes are manifold. Superintendents, pupils, and graduate nurses alike have their peculiar grievances, and the remedies proposed are drastic, going somewhat further than the foremost reformers in England consider necessary—at least, just at present. They are as follows: To (1) send forth fewer graduates from the large training schools; (2) prevent the granting of certificates and graduation of nurses from all schools which fail to come up to a fixed standard; (3) prevent the formation of all new schools that are not formed expressly to supply a need felt in that community; (4) open up a wider field for the graduate nurse.

The writer's idea is to nurse the hospital by graduate nurses, and to make the pupils pay for the privilege of studying in the schools connected with these hospitals. This, of course, at once opens up a wider field for the trained nurse, and the extra cost falls not on the hospitals, but on the pupil nurse, who will pay "for her board, for her laundry, and her room. Her uniform, if such be worn, will be provided by her; the text-books, the instruments, everything will be provided, not by the school, but by the pupil; and, in addition, she will pay for the privilege of being allowed to nurse in the wards of the hospital and for the theoretical and practical training she receives."

The advantages that the pupil receives will be that she has careful and thorough instruction in nursing, and that her studies will not be interfered with by any work that is not necessary to her training during her noviciate.

It needs no Solomon to discern the result of such regulations. In the event of their becoming general nursing for the future must fall exclusively into the hands of those who have money to pay for a long and costly training; the fees for nursing the sick must in consequence become heavier, whilst the public must learn to rely more on the willing hearts and unskilled hands of the "born nurses," who—and let mankind be duly thankful for it—are as, if not more, plentiful amongst the poor as amongst the rich; in other words, the public must largely depend on untrained nursing!

Mr. Edward C. Thayer's memorial to his two sisters has taken the form of a handsome home for the residence of the nurses and pupils attached to the City Hospital, Worcester, Mass. It was dedicated on June 15th, 1898, and has cost 50,000 dollars. It is built of red brick ornamented with stone facings, and the basement is of granite; the interior is of oak, and each sleeping apartment is furnished with the same wood excepting that the bedstead is of iron. It is fitted with electric light and all modern conveniences. In the basement are placed the heating and ventilating apparatus, trunk and store rooms. On the ground floor are the matron's suite of rooms, three class-rooms capable of being thrown into one large one, reception-room, parlour, and library; on the first floor are nine bed-rooms; on the second floor are nineteen bed-rooms, a large sitting-room, and three sick rooms; on the third floor are twenty-one bed-rooms, and above is an unfurnished attic.

A Hawaiian branch of the Red Cross Society is in process of organisation. The local secretary is Miss Emily Forster Day and the president and vice-president are Mrs. Sewall, wife of the American Minister at Honolulu, and Mrs. Dole, wife of the President of Hawaii. The National Red Cross Society has recognised and welcomed the new branch.

Miss Louise Darch, late superintendent of the New York City Training School for Nurses, Blackwell's Island, whose resignation from ill-health took place some time ago, is rapidly regaining strength, and it is reported that she and Miss Diana Kimber, her successor, are contemplating a trip abroad together.

### A Pro's Resolve.*

I like to feel that I've begun
 The life I've longed to lead,
But little did I think it one
 Where so much strength I'd need.
Till late at night, from early morn,
 I toil, and toil, and toil;
And oh! the sister's look of scorn
 Because the charts I spoil.
"Reports are incomplete," she says,
 "The work not neatly done";
But these are only early days,
 Wait till my training's done.
I'll toil and toil, and every morn
 Resolve to mend my ways,
And soon the sister's look of scorn
 Will change to one of praise.
No more at eight the milk forgot,
 No "hammering" required,
The fomentations shall be hot,
 Tho' I be fagged and tired.
And sister's hair shall not be grey,
 Nor shall she have to wait
So very long before the day
 That seals my happy fate.
I mean the day that comes at last
 To pros who persevere,
When, all exams. with credit passed,
 Their sisters' hearts they cheer.

* An Answer to "A Sister's Lament."

### St. Luke's Home, Vancouver, B.C.

June 21st saw the completion and opening of the "Sons of England" ward of the St. Luke's Home, Vancouver, a Jubilee memorial, where Englishmen can be received and nursed for a fixed sum of 5 dols. a week. It was opened free of debt, and fully furnished. The ward is a bright airy room with three beds, and opening out of it are a nurses' sitting-room and a small operating-room, forming quite a compact little flat. After a brief opening address and prayer by Mr. Fiennes-Clinton, their chaplain, the grand ceremony of hanging the autograph portrait presented by Her Majesty the Queen took place amidst loud cheers and the singing of the National Anthem. The portrait has been handsomely framed, and is, as far as we know, the first one hung in a Canadian hospital, an honour of which the St. Luke's Home is proud. The "Daughters of England" presented the linen for the ward.

After the opening ceremonies the "Sons and Daughters of England" were entertained by Sister Frances and the nurses of the home, and a very enjoyable evening was spent.

### Minor Appointments.

The Sisters' Hospital for Persons Suffering from Infectious Diseases, St. Albans.—Miss Sarah Dutton, who was trained at Chester General Infirmary for three years, and at the Monsal Hospital, Manchester, for two years, was appointed Matron of the above on the 16th inst. She was hospital nurse for two years at Chester General Infirmary, and charge nurse for sixteen months at the Eastern Fever Hospital, Homerton, N.E.

Kent County Asylum, Chartham, Canterbury.—Miss Edith P. Dring was appointed Assistant Matron of this asylum on the 22nd inst. She was trained at St. George's Hospital, London, and was afterwards charge nurse at the Royal Infirmary, Windsor.

York County Hospital. — Miss J. Watts has been appointed Home Sister to the York County Hospital. She was trained for three years at the Royal Hants County Hospital, and has recently gained a three years' certificate with credit.

### "THE HOSPITAL" CONVALESCENT FUND.

We tender our grateful thanks to Miss Olive Richards for her kindly gift of 5s. to the Convalescent Fund.

### Everybody's Opinion.

[Correspondence on all subjects is invited, but we cannot in any way be responsible for the opinions expressed by our correspondents. No communication can be entertained if the name and address of the correspondent is not given, as a guarantee of good faith but not necessarily for publication, or unless one side of the paper only is written on.]

### LADIES V. GENTLEWOMEN.

"Lear" writes: I should like to say that I quite agree with "Nurse," for I have always defined a gentlewoman as a woman of refined and gentle ways and a pure mind, and I maintain that every woman may be a gentlewoman if she likes. Now a lady, I think, means birth and education —not necessarily the latter—and it certainly is possible for a woman to be a lady without being a gentlewoman. That I have proved. If "Matron" advertised for a nurse and put "Lady" like that, she, perhaps, would not be so vexed with the answers she gets, as surely then only ladies would apply.

### NURSING AT THE COAST HOSPITAL.

An "Old Nurse" writes: In looking over The Hospital Mirror for May 28th I am more than surprised that "Nurse M. P.," so long in Sydney, should disagree with your report in "The Mirror" of May 21st in regard to Little Bay State Hospital, Sydney. About a year ago Mr. Griffith, M.P., had a great fight in the House of Parliament to get nurses' working hours placed under the Factory Act, but failed. There was much debating and comparing of notes in regard to health and mortality of nurses in general, and medical members tried to show that nurses' working hours, health, and hospital comforts were in every way satisfactory, and that frequent changing of the nursing staff would mean danger to the patients; indeed medical gentlemen on the staff of Prince Alfred Hospital said "Nurses' lives bordered on the side of luxury." Then the truth came out that the first object was the saving of money (the usual object out here in a good cause), and the Government, after a hard fight, becoming convinced that there was room for improvement, decided about six months ago to put their own house in order. It began by increasing the staff at Little Bay Hospital. "M. P." is quite right in saying that private nurses as a rule in Sydney have a hard fight to keep body and soul together. Many nurses have not the ability to do any and every thing that turns up, and consequently they fail.

### REAL GENTLEWOMEN.—BLOOD OR PRINCIPLES?

"Brighouse" writes: Neither birth, education, or the texture of one's clothes determine the real gentlewoman. The qualities which go toward the making of gentlewomen in the true sense of the word can only be cultivated and developed by the individual herself. Real gentlewomen need not necessarily be of gentle birth. We have only to read our daily newspapers to learn that. Their pages teem with the doings of the "upper ten," which we would blush to see enacted in our midst. Real gentlewomen are recognised by their lives and not by their dress. By their actions and not by their speech. By their natural and unassuming manner, and by their strength to crush the evil and uphold the right, even though it may mean harm for them. Real gentlewomen are modest and just, refined, and sincere, who would rather soil their hands with honest labour than soil their tongues with lying. If needs be they will dress in a cotton garb, but would scorn to clothe themselves in a garment of deceit and dishonour. If she cannot win success by honesty she will lose it rather than gain it by trickery. Whatever her aim may be she works up to it, always thinking and acting as a gentlewoman. Real gentlewomen scorn to slander and speak evil of anyone, and would loathe to become like the type which Ruskin compares "to a serpent that crawls along on its belly." Real gentlewomen do not prevaricate, but speak the truth at any cost. They are just and merciful, and do not crush the weak. Real gentlewomen are not recognised by their grammatical construction of sentences, but by their refinement of speech. They may make grammatical mistakes, but will not use slang. Real gentlewomen will not consciously hurt people's feelings by uttering unkind remarks, but are the defenders and protectors of the weak and respect the aged. Despite the culture and progress we have arrived at, we live in an age of vulgarity. Real gentlewomen do not use the most high-sounding words in speech. Their language is simple and unaffected. Neither do they introduce words or sentences of French, German, Latin, or any other foreign tongue in their conversation, making, as it were, sandwiches of their writings and discourses. It matters not where we were born—in a palace or a cottage —rich or poor. It depends upon ourselves whether we are real gentlewomen. Riches can no more determine it than education or the chance of our birth. Whether we succeed or fail in our attainments let our characters be high and our lives clean. Let us take for our motto the golden saying of Ruskin, "Strive rather to be, than to seem," and then in truth we will undoubtedly be real ladies and gentlewomen, and help to make the world we live in a much happier one.

### A CANADIAN NURSE'S HOLIDAY.

Sister Frances, of the St. Luke's Home, Vancouver, writes from Halifax, N.S., under date August 8th, 1898: Here I am, after seven years' steady work, on a holiday. I left Vancouver on July 11th for Montreal—a week's journey —where I had to remain for a night, as, owing to there being no Sunday traffic, there is one day a week when trains do not leave, and this happened to be the day for no train to Halifax, so I went to an hotel and registered. After dinner I ran off to Mr. Wood, the rector of St. John the Divine, and also to see Sister Elizabeth, of St. Margaret's, and they invited me to spend the next day, Sunday, so I had a pleasant enough day. The St. Margaret's Home for chronic cases is a beautiful one, with every possible luxury and comfort. It was presented to the sisters some years ago by a rich Montreal gentleman, who is still very generous to them and keeps it in repair. Then on to Halifax, which is a twenty-four hours' journey through very lovely scenery, although after the magnificent Rockies I cannot do more than say it is pretty. At Halifax I am enjoying a total rest and change; nothing to remind me of hospital life. The institutions at Halifax are very fine. I have visited the Deaf and Dumb Asylum, and it is a splendid place, at which about 90 children are received and taught. Huge corridors and stairways and large windows give one an idea of space and air not expected. One great means of saving of time I saw, and it is new to me. Each room belonging to the staff has a telephone which communicates with the other members of the staff, so you can call up any particular room in the building. The matron had one for her kitchen staff. These telephones are not for outside use, and it seemed a much quicker arrangement than speaking tubes, and besides they had the city telephone for outside purposes. The old ladies' home, too, is very large and accommodates 36 inmates, each having a separate bed-room. Here, again, I found large airy corridors, so that the inmates can walk up and down and visit each other without the fatigue of going to the general reception-room. This is hardly a charitable institution, as each lady pays a small sum yearly. It is very small, but it just misses the word charitable. There is a large endowment, and so the institution is quite above want. Halifax is a city of churches, only one I am sorry to say with open doors daily. The music is very beautiful, and Sunday worship is strictly observed; the congregations are good. I should say overworked nurses needing holidays would like it very much at Halifax. It is direct from London and a cheap place to stay in, at least cheap for the colonies. The sea voyage, too, would be such a boon. Should a nurse reading this think she would like to try a holiday she should make for the clergyman of whatever church she belongs to, and he would advise her where to board. I believe it can be got nicely for 10s. to 12s. per week in a good locality, and the hotels would not be very much, about ten dollars per week. There are heaps of excursions and picnics, and for a short holiday I think the change would be more beneficial than one in a non-English speaking place. I have made the acquaintance of several clergymen. One, Mr. Lemoine, is from Canterbury, and his curate, Mr. Symmonds, from Warminster. Either of these gentlemen would, I am sure, help anyone with advice. The public gardens here are very beautiful; I saw there a century plant in bloom, and there is a very good public library.

### For Reading to the Sick.

### "THE WILL OF GOD."

### Verses.

We abide
Not on this earth; but for a little space
We pass upon it; and while we so pass,
God through the dark hath set the Light of Life,
With witness of Himself—the Word of God,
To be among us Man, with human heart
And human language—thus interpreting
The One Great Will incomprehensible,
Only so far as we in human life
Are able to receive it. —*H. Hamilton King.*

God's voice is of the heart—I do not say
All voices therefore of the heart are God's;
And to discern the voice amidst the voices
Is that hard task that we are born to. —*Clough.*

God hath now sent His living oracle
Into the world to teach His final will. —*Milton.*

How very good is God! that He hath taught
To every Christian that can hear and see
Both what he is and what he ought to be,
And how and why the saints of old have fought.
Whate'er of truth the antique sages sought,
And could but guess of His divine decree,
Is given to faith affectionate and free,
Not wrung by force of self-confounding thought.
Is the book finished? May not God once more
Send forth a prophet to proclaim His laws
In holy words not framed by human lore?
—*H. Coleridge.*

### Reading.

"Doing the will of God from the heart."—*Eph.* vi. 6.

This short sentence—eight words in the English, seven only in the Greek—is a wonderfully comprehensive account of the action of the spiritual life. Take it word by word, and every detail in it is a great principle, meant to underlie a most happy experience and practice. "Doing" is its first word; doing, as against dreaming; doing, in the sense of a genuine obedience, and not merely an approval, a recognition, of what claims to be obeyed. "Doing the will of God" is its next word, reminding the Christian that he is indeed not his own, that he exists for Another, for his Maker and Redeemer, and that his own being will never work aright, will never fulfil its true "law," will never rest, out of the line of the will of Him Who has made him, has remade him, owns him altogether, and purposes to use him. "Doing the will of God from the heart," or more precisely, "from the soul," is its last word; a word which conveys at once precept and promise; bids the man seek such a "doing" as shall be not friction and fatigue, but a matter of strong, warm interest and willingness, "not a sigh, but a song"; and by thus bidding him seek, assures him that he shall find; tells him that such a doing is divinely possible in the life of grace, no daydream, but a living and practical reality for "the children of the day." "The will of God!" Let us, to animate and endear every thought of it, remind ourselves often of its blissful purposes. If it wills for us immediately toil and trial, contradictions, disappointments, tears—as it sometimes does, as it once did for our Lord and Life—what does it always will ultimately, and with infinite skill and power, to attain its end? It wills, He wills, "that not one of His little ones should perish." He wills "that everyone that seeth the Son and believeth in Him should have everlasting life, and be raised up again by Christ Jesus at the last day." He wills "our sanctification." He wills, as His Son wills, that they whom He has "given" to His Son should "be with Him where He is to behold His glory."—*H. C. G. Moule.*

### Notes and Queries.

*Free Training.*

(199) Is there any lying-in hospital where a free training is given for six months' services? The inquiry is on behalf of a well-educated young lady, age 27, who has had six months' preliminary training in a cottage hospital.—*Buxton.*

There is no lying-in hospital that gives free training.

*What Constitutes a Certificate?*

(200) This hospital contains 17 beds. We get male and female medical and surgical work, also a great many serious accidents. The training is so good that I would like to give a certificate to my probationers. How can I manage it? Is it a question of lectures from the medical officers or from myself? What constitutes giving a certificate for training?—*K. B. M.*

In order that your institution may be reckoned as a training school by the Local Government Board it must maintain a resident physician or house surgeon. If this be so, you can arrange courses of instruction for your nurses, and at the end of three years your committee can grant them certificates qualifying them for offices under the Poor Law, &c. Write to the secretary of the Local Government Board first and obtain the Board's recognition of your institution as a training school.

*India.*

(201) Is there not an association for supplying English-trained hospital nurses for Europeans in India? If so, will you kindly send me the address of the secretary.—*M. W. C.*

The Up-Country Nursing Association for Europeans in India. Hon. Secretary, Major-General J. Bonus, The Cedars, Strawberry Hill, W.

*Medal for Mental Nursing.*

(202) I am a fully-qualified nurse, with ten years' experience in nursing. Will you tell me how long it will be necessary for me to remain in an asylum in order that I may apply for the medal for mental nursing?—*A. H.*

We presume that you mean the medal of the Medico-Psychological Society. One year is enough, as you are already trained in sick nursing. A course of instruction is necessary during that year, and you should apply to the Registrar, 11, Chandos Street, W., for particulars.

*Monthly Nursing.*

(203) Can you tell me if I can possibly get trained in monthly nursing free, or if there is any society for helping a gentlewoman towards paying the expenses of such. If not, where is the best and least expensive training to be had?—*L. E. H.*

The cheapest course of maternity training is offered by the East-end Mothers' Home, 396, Commercial Road, E., the inclusive cost of which is £15 15s. If you have no capital, and are desirous of this kind of work, your best plan appears to be to obtain a training as a sick nurse. You could then save money to pay your fees.

*The "Asylum News."*

(204) Will you kindly tell me how to get a copy of the *Asylum News?* I have tried the local booksellers, and none have succeeded in getting it for me. Is it a weekly publication, and what is the price per copy?—*Cozen.*

See advertisements in THE HOSPITAL from time to time.

*Dispenser's Post.*

(205) Can you tell me the best way of obtaining a dispenser's post? I have the Apothecaries' Hall certificate, and have had two years' experience (one year assisting at a hospital).—*Dispenser.*

Advertise and see advertisements. There was a dispenser required amongst the "Appointments Vacant" in *London* for August 11th.

*Training in Children's Hospital.*

(206) Kindly let me know of children's hospitals in which a young friend of mine could be trained. She is 20, and requires a salary. Would infirmary work be more advantageous?—*Nurse Martin.*

Consult "Burdett's Official Nursing Directory," where you obtain full details of training and salaries of all the schools. If you call at this office you may see a copy. The price is 5s., from the Scientific Press, London.

*Training Under a Lady Dispenser.*

(207) Would it be possible to become resident pupil under a lady dispenser, preferably in London?—*Puella.*

It should not be impossible, and your best plan to find such a post would be by advertisement.

*The Children's Nurse.*

(208) Can you give me the address of an institution where girls are trained specially as children's nurses?—*T. S. S., Edgbaston.*

The Liverpool Ladies' Sanitary Association, 8, Sandon Terrace, Liverpool.

*Elementary Nursing.*

(209) Is there any nursing home or infirmary in West London where ladies who are training as Church workers might go two or three days in the week and receive some elementary instruction in nursing?—*The Sister in Charge, S. Gabriel's House, 12a, Tavistock Crescent, W.*

Courses of lectures on nursing are arranged in connection with the various polytechnic institutions, and by the Young Women's Christian Association, offices 25 and 26, George Street, Hanover Square, W.

*London Obstetrical Society.*

(210) Would you kindly tell A. S. if she can obtain a certificate from the London Obstetrical Society without being trained; if so, how to set about it, and the cost.

The London Obstetrical Society holds examinations four times a year, for which the fee is £1 1s. Candidates must be 21 years of age, and have attended a lying-in hospital or charity for not less than three months, and have attended personally 25 labours and a course of theoretical teaching. The cheapest course is that of the East-end Mothers' Home for £15 15s., including all but examination fee.

